# Changes in Serum Iron and Leukocyte mRNA Levels of Genes Involved in Iron Metabolism in Amateur Marathon Runners—Effect of the Running Pace

**DOI:** 10.3390/genes10060460

**Published:** 2019-06-15

**Authors:** Agata Grzybkowska, Katarzyna Anczykowska, Wojciech Ratkowski, Piotr Aschenbrenner, Jędrzej Antosiewicz, Iwona Bonisławska, Małgorzata Żychowska

**Affiliations:** 1 Department of Biochemistry, Faculty of Physical Education, Gdansk University of Physical Education and Sport, 80-336 Gdansk, Poland; agata.p3@gmail.com (A.G.); kasia.anczykowska@gmail.com (K.A.); jant@gumed.edu.pl (J.A.); 2 Department of Management in Tourism and Recreation, Faculty of Tourism and Recreation, University of Physical Education and Sport, 80-336 Gdansk, Poland; maraton1954@o2.pl; 3 Department of Life Sciences, Faculty of Physical Education, Gdansk University of Physical Education and Sport, 80-336 Gdansk, Poland; sqarko@gmail.com; 4 Department of Anatomy and Anthropology, Faculty of Physical Education, Gdansk University of Physical Education and Sport, 80-336 Gdansk, Poland; iwonabonislawska@gmail.com

**Keywords:** iron metabolism, ferritin, gene expression, marathon runners, *PCBP1*, *PCBP2*, *TFRC*

## Abstract

Iron is essential for physical activity due to its role in energy production pathways and oxygen transportation via hemoglobin and myoglobin. Changes in iron-related biochemical parameters after physical exercise in athletes are of substantial research interest, but molecular mechanisms such as gene expression are still rarely tested in sports. In this paper, we evaluated the mRNA levels of genes related to iron metabolism (*PCBP1*, *PCBP2*, *FTL*, *FTH*, and *TFRC*) in leukocytes of 24 amateur runners at four time points: before, immediately after, 3 h after, and 24 h after a marathon. We measured blood morphology as well as serum concentrations of iron, ferritin, and C-reactive protein (CRP). Our results showed significant changes in gene expression (except for *TFRC*), serum iron, CRP, and morphology after the marathon. However, the alterations in mRNA and protein levels occurred at different time points (immediately and 3 h post-run, respectively). The levels of circulating ferritin remained stable, whereas the number of transcripts in leukocytes differed significantly. We also showed that running pace might influence mRNA expression. Our results indicated that changes in the mRNA of genes involved in iron metabolism occurred independently of serum iron and ferritin concentrations.

## 1. Introduction

Iron is essential for physical activity due to its role in energy production pathways and oxygen transportation via hemoglobin and myoglobin [1]. Athletes are considered to be at greater risk of iron deficiency than the general population, although supportive data are inconclusive [2,3]. In one particular study, iron deficiency was found in 1.6% of recreational runners, but iron overload was found in 15% of the male participants [4].

Recently, research interest in iron metabolism during and after exercise has grown because physical activity can affect iron and iron-regulatory protein status in many ways, such as by inducing oxidative stress and inflammation [5,6,7,8,9,10]. In the case of intense running efforts, foot strike causes hemolysis as an additional factor that contributes to disordered iron metabolism [11,12]. Terink et al. [13] reported that most of the studies related to iron metabolism were conducted on well-trained athletes, mainly during short and intensive efforts. Additionally, the changes in iron metabolism were determined only on protein concentrations in plasma or serum and usually showed an increase in blood ferritin values, although the findings were conflicting. To the best of our knowledge, there are no data focused on the effect of endurance exercise on the mRNA of genes related to iron metabolism.

The popularity of marathon running has increased in recent years especially in amateurs of different ages, sex and physical capabilities. It seems that the runner’s age, running speed and level of adaptation to training are main influences of the physiological response to physical exercise. However, published studies also have some contradictory findings. For example, Jastrzębski et al. [14] reported that during a 100 km run, muscle and liver damage was age but not pace-dependent while negative metabolic changes were independent of age. 

The aim of our study was to examine the changes in serum iron and ferritin concentrations together with the changes in leukocyte mRNA levels of genes encoding proteins involved in iron metabolism i.e., *PCBP1* (poly(rC) binding protein 1), *PCBP2* (poly(rC) binding protein 2), *FTH1* (ferritin heavy chain 1), *FTL* (ferritin light chain) and *TFRC* (transferrin receptor). The expression of these genes is expected to be affected by marathon running as the proteins they encode are involved in exercise-induced oxidative stress and inflammation [15,16,17]. We also aimed to evaluate the relationship between changes in gene expression and baseline serum iron and ferritin concentrations, and runner pace, during a run. We hypothesized that marathon running will induce an increase in the mRNA levels of genes associated with iron metabolism similarly to serum changes and that these changes will be pace-dependent.

## 2. Materials and Methods

### 2.1. Characteristics of the Subjects and Baseline Laboratory Parameters

A total of 28 healthy young men who reported regular physical activity involving a running program participated in our study. All participants were asked to refrain from changing their diet and to avoid nicotine and alcohol use, for one month prior to undertaking the study marathon run (42.2 km) at an athletic stadium (Gdansk University of Physical Education and Sport, Gdansk, Poland). The run was completed by 26 of the 28 participants, and a further two subjects were excluded as they had mRNA levels that were far from the average. Anthropometric data for the 24 included subjects are shown in Table 1. All the subjects were informed of the purpose of the study and the possible risks involved before giving written consent. The study was approved by the Bioethics Committee for Clinical Research at the Regional Medical Chamber in Gdansk (NKBBN/448/2016). The principles of the Helsinki Declaration were respected.

### 2.2. Experimental Procedure

Venous blood was collected and serum was obtained from Vacutest^®^ Clot Activator tubes (Vacutest KIMA, Arzegrande, Italy) at four time points: before the run (pre-race), immediately after finishing the run (post-race), 3 h after the run (3 h post-race) and 24 h after the run (24 h post-race). The blood samples were analyzed for blood morphology, and serum concentrations of iron, ferritin, uric acid, creatinine kinase and C-reactive protein (CRP) at an accredited laboratory (Uniwersyteckie Centrum Kliniczne, Gdansk, Poland). Right before the run, the subjects’ body weight, height, body mass index (BMI) and percentage of body fat (PBF) were determined using InBody 720 (Biospace Co., Ltd., Seoul, Korea) [18].

To assess gene expression, a further 2 mL of venous blood was collected using vacutainers spray-coated with K_3_EDTA as an anticoagulant at the same four time points. The collected blood was mixed within 15 min with Red Blood Cell Lysis Buffer (RBCL) (A&A Biotechnology, Gdynia, Poland) and incubated on ice for at least 15 min. The samples were then spun at 3000× g at 4 °C for 10 min. The resulting pellet was washed again with the hemolysis buffer and the remaining white blood cells lysed using Fenozol (A&A Biotechnology, Gdynia, Poland), and immediately after stored at −20 °C for up to four months, with no freeze–thaw cycles.

### 2.3. RNA Extraction and Reverse Transcription

Isolation of total RNA was carried out by the modified Chomczynski and Sacchi method [19]. White blood cells diluted in fenozol were thawed at 50 °C for 5 min. Then 200 µL of chloroform (POCH, Gliwice, Poland) was added and the suspension was shaken. Samples were then centrifuged at 10,000 *g* for 30 min at 4 °C. The aqueous phase was collected and mixed with 500 µL of isopropanol (POCH, Gliwice, Poland) and left for at least 30 min to precipitate RNA. Samples were again spun at 10,000 g for 15 min at 4 °C. The aqueous phase was disposed, and the remained pellet was washed 2 times in 1 mL of 75% ethanol at 7500 g at 4 °C. After drying, the pellet was resuspended in 20 µL of PCR grade water. During the optimization period for tested genes, gel electrophoresis has been performed to check for the quality and integrity of RNA. RNA concentration and purity were determined by spectrophotometer (Multiskan Sky Microplate Spectrophotometer, ThermoFisher Scientific, Warszawa, Poland) by absorbance at UV 260/280, and a ratio >1.7 was accepted as pure RNA suitable for further analysis. RNA was then reverse transcribed to cDNA in Eppendorf Mastercycler Gradient 5331, using 0.2 µM oligo(dT) and a Transcriptor First Strand cDNA Synthesis Kit as per the manufacturer’s instructions (Roche, Warszawa, Poland). For the analysis 1000 ng of RNA has been used. Thermal conditions used for this step were as follows: Incubation—60 min at 50 °C—followed by inactivation—5 min at 85 °C. Prepared samples were frozen immediately after the reverse transcription and then stored at −20 °C for up to one month, with no freeze–thaw cycles. For gene expression analysis, the obtained cDNA was diluted 10 times, just before the qRT-PCR step.

### 2.4. Quantitative Polymerase Chain Reaction Assay

Quantitative real-time polymerase chain reaction (qRT-PCR) analyses were carried out on six genes of particular physiological significance in the context of iron metabolism. The AriaMx Real-Time PCR System (Agilent Technologies, Warszawa Poland) and FastStart Universal SYBR^®^ Green Master (Rox) (Roche, Warszawa, Poland) were used, according to the manufacturer’s protocol, on 96-well PCR plates in triplicate for each sample. 2 µL of diluted cDNA was used for qRT-PCR. The thermal cycling conditions comprised an activation step: 95 °C for 10 min followed by 40 cycles of annealing; and an extension step: 95 °C for 15 s and 60 °C for 1 min. Additionally, the melt curve analysis was performed for each reaction. *TUBB* (tubulin beta class I, NM_001293213) was chosen experimentally and used as the reference gene. The relative mRNA expression of *PCBP1* (NM_006196), *PCBP2* (NM_001128913), *FTH1* (NM_002032), *FTL* (NM_000146), *CAT* (catalase, NM_001752) and *TFRC* (NM_001128148) was calculated using qRT-PCR. The primer sequences were designed by the authors using the Primer3 Web tool. In silico specificity screen has been performed using USCS genome browser. The primers were then ordered from Genomed, Warszawa, Poland. Primer sequences (5’-3’), were:

TUBB 

Forward primer: TCCACGGCCTTGCTCTTGTTT

Reverse primer: GACATCAAGGCGCATGTGAAC

PCBP1

Forward primer: AGAGTCATGACCATTCCGTAC

Reverse primer: TCCTTGAATCGAGTAGGCATC

PCBP2

Forward primer: TCCAGCTCTCCGGTCATCTTT

Reverse primer: ACTGAATCCGGTGTTGCCATG

*FTH1*

Forward primer: TCCTACGTTTACCTGTCCATG

Reverse primer: CTGCAGCTTCATCAGTTTCTC

FTL

Forward primer: GTCAATTTGTACCTGCAGGCC

Reverse primer: CTCGGCCAATTCGCGGAA

CAT

Forward primer: GATGGACATCGCCACATGAAT

Reverse primer: AAGATCCCGGATGCCATAGTC

TFRC

Forward primer: TGCAGCAGTGAGTCTCTTCA

Reverse primer: AGGCCCATCTCCTTAACGAG

### 2.5. Statistical Analysis

#### 2.5.1. Serum Parameters

Whole blood measurements were corrected for plasma volume shift using the Dill and Costill equation [20]. The normality of the distributions was checked for all parameters using the Shapiro–Wilk test. Values were compared statistically using the one-way analysis of variance (ANOVA) test followed by Tukey’s multiple comparisons test for parametric data, and Kruskal–Wallis test followed by Dunn’s multiple comparisons test for nonparametric data.

#### 2.5.2. mRNA Levels

Relative mRNA expression was determined using the Schmittgen and Livak delta delta C_t_ method [21] in Microsoft Excel (2017). The mRNA levels of the tested genes were described as the differences in the cycle threshold value normalized to the *TUBB* mRNA level, i.e., ΔC_T_ = C_T_ of gene—C_T_ of *TUBB.* All statistical analyses were performed using GraphPad Prism 8.0 (GraphPad Software, Inc., La Jolla, CA, USA).

Relative mRNA expression data were linearly transformed and then the normality of the distribution was checked with the Shapiro–Wilk test. Results were analyzed using the Wilcoxon matched pairs [22] test for nonparametric variables. A p-value of <0.05 was considered significant. 

## 3. Results

### 3.1. Blood Morphology 

Statistically significant changes were observed in all white blood cells, uric acid and creatine kinase immediately after the run. Changes in other laboratory parameters occurred 3 h post-race, except for CRP which was significantly elevated after 24 h post-race. All values were corrected for changes in plasma volume (%Delta PV). All results are shown in Table 2.

### 3.2. Serum Ferritin and Iron Concentrations

**Figure 1:**
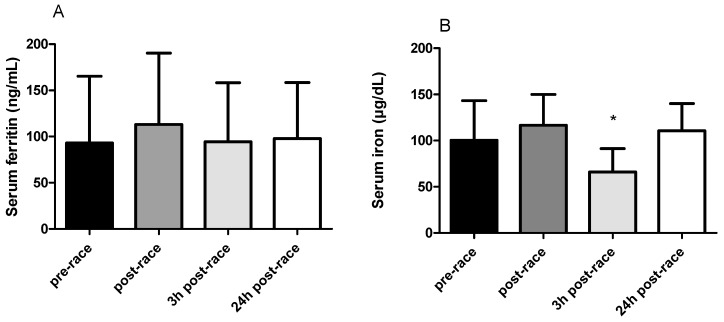
Serum ferritin (**A**) and iron (**B**) concentrations at different time points in the 24 participants. Values are presented as mean ± SD. * *p* < 0.05 compared to the pre-race value.

No significant changes in ferritin concentrations were observed immediately after the run or during the recovery period, compared with baseline. There was a slight tendency to an increase in ferritin immediately after the run compared with the pre-race rest value (113.1 versus 93 ng/mL, respectively). However, ferritin at 24 h after the run was essentially unchanged from the pre-race value (97 versus 93 ng/mL, respectively). The same direction of changes was observed in serum iron, but at 3 h after the run there was a significant decrease compared with baseline values (66.1 versus 102.3 µg/dL, respectively; *p* = 0.002). Between 3 h and 24 h post-race, serum iron increased and had returned to baseline by 24 h (Figure 1).

### 3.3. Effect of Exercise on mRNA Levels of Selected Genes 

**Figure 2:**
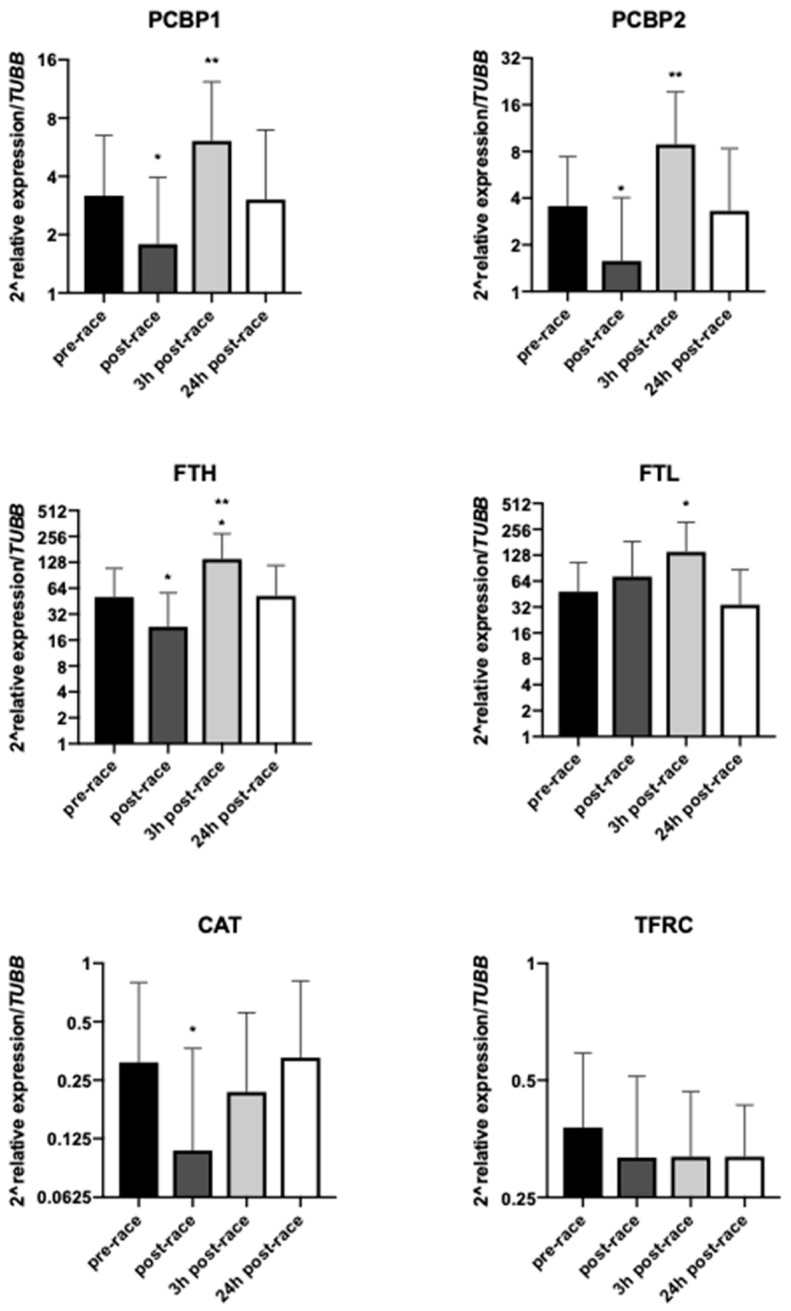
mRNA levels of selected genes at different time points (*n* = 24). Values are presented as mean ± SD. * *p* < 0.05 compared to the pre-race value and ** *p* < 0.05 compared to the post-race value.

Out of six genes tested, five were down-regulated at the end of the race compared with baseline, with the differences for *PCBP1, PCBP2, FTH* and *CAT* achieving statistical significance (*p* = 0.0359, *p* = 0.0443, *p* = 0.0158 and *p* = 0.0182, respectively) (Figure 2). There was a trend for up-regulation in *PCBP1* and *PCBP2* (*p* = 0.0826 and *p* = 0.2435, respectively)*,* and a significant up-regulation in *FTH* and *FTL* genes (*p* = 0.0056 and *p* = 0.0064, respectively) at 3 h after the marathon run. The mRNA levels of all genes except for *TFRC,* which remained insignificantly decreased, returned to baseline levels at 24 h after the run.

### 3.4. Relationship Between Baseline Levels of Serum Iron and Ferritin, and Changes in mRNA Levels with Exercise

There were no statistically significant differences in mRNA levels at any time point in participants with baseline serum iron concentrations below (serum iron ≤ 105 µg/dL) and above (serum iron >105 µg/dL) the median baseline value (data not shown). There were also no statistically significant differences in mRNA levels in participants with baseline serum ferritin concentrations below (serum ferritin ≤ 78.08 ng/mL) and above (serum ferritin > 78.08 ng/mL) the median baseline serum ferritin value (data not shown).

### 3.5. Effect of Running Pace on mRNA Levels of Selected Genes

**Figure 3:**
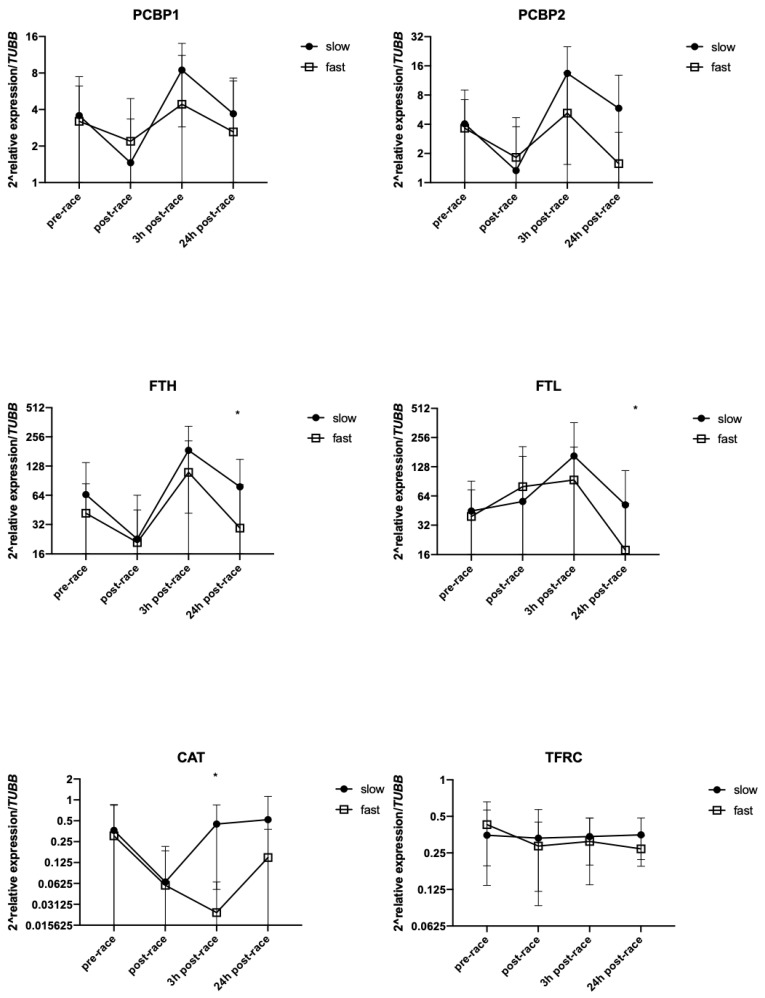
Changes in mRNA levels between slow and fast runners (*n* = 12 per group). Values are mean ± SD. * *p* < 0.05 for comparison between two groups.

To determine if the running pace had any effect on gene expression, the participants were divided into two groups (slow and fast) by the median split. The characteristic of two groups is shown in Table 3. A significant difference between groups was observed for pace (*p* = 0.0001), BMI (*p* = 0.006) and age of the participants (*p* = 0.0001). The mean ± SD pace value in the slow group was 10.0 ± 0.5 km/h and in the fast group was 12.2 ± 0.7 km/h (*p* < 0.0001). The mRNA levels of the genes tested in these two groups are shown in Figure 3.

The direction of change in *PCBP1*, *PCBP2* and *FTH* gene expression were the same i.e., a decrease immediately after the race and a statistically significant increase 3 h post-race. At 24 h post-race, the values returned almost to baseline (Figure 3). *FTL* mRNA levels were more stable than *FTH* mRNA levels between the end of the run and 3 h post-race. However, similarly to *FTH*, a significant difference in *FTL* mRNA levels between groups was observed 24 h after the race (*p* = 0.0245 and *p* = 0.0128, respectively). The slow group presented with higher levels than the fast group. The opposite changes were observed in *CAT* mRNA levels at 3 h post-race. In the slow group *CAT* mRNA levels dropped, while they increased in the fast pace group (*p* = 0.0017).

## 4. Discussion 

The results of this study did not confirm our hypothesis associated with serum iron, ferritin and expression of genes involved in iron metabolism. Serum ferritin concentrations remained almost unchanged at all time points. Iron status immediately and 24 h after completion of a marathon also did not differ from baseline but there was a significant decrease 3 h after the run. Moreover, changes in iron and ferritin did not correlate with each other (data not shown). Interestingly, a significant decrease in *FTH, PCBP1, PCBP2* and *CAT* mRNA was observed immediately after the run, and a significant increase in *PCBP1, PCBP2, FTH* and *FTL* mRNA was seen at 3 h after the run. *TFRC* mRNA remained unchanged. Furthermore, changes in serum indicators and gene expression in leukocytes occurred at different time points.

### 4.1. Changes in Serum Iron and Ferritin Concentrations

Interindividual variability was observed in baseline serum iron (39–196 µg/L) and ferritin (8.2–367.9 ng/mL) concentrations. The literature on the changes in iron status induced by endurance exercise is equivocal. A decrease in serum iron concentrations 24 h after a marathon was reported by Roecker et al. [23], by Terink et al. [13] after repeated walking and by Chiu et al. [24] after an ultramarathon. On the other hand, an increase in iron concentrations was reported by Peeling et al. [25] after a triathlon and by Buchman et al. [26] after a marathon. According to Terink et al. [13], these differences could be associated with changes in plasma volume and whether this parameter was taken into consideration before the analysis of the results. We corrected for changes in plasma volume and our findings were similar to those reported by Duca et al. [27]. These authors found no change in serum iron or ferritin at 24 h after a half-marathon. Similar findings in serum iron and ferritin concentrations at 24 and 48 h after a marathon were also reported by Weight et al. [28]. Unfortunately, there appear to be no studies in which ferritin and iron concentrations were determined at 3 h after exercise. It is important to note that at this time point increased serum hepcidin was observed [25], and it can be assumed that this would be accompanied by a drop in serum iron, which is consistent with our data. At 24 h after the marathon, basal values had been attained in the participants of our study. In contrast to our results, at the same time point (1 day after prolonged walking) Terink et al. [13] reported decreased iron concentrations. These authors also corrected their results for the change in plasma volume. Lack of a significant correlation between serum ferritin and iron was observed earlier by Galanello et al. [29]. These authors reported that after a stressful event such as a marathon run, the serum ferritin concentrations could not accurately reflect body iron status. Moreover, the observed nonsignificant changes in ferritin concentrations at 24 h after a marathon are in agreement with data reported by Terink et al. [13]. Indirectly, the nonsignificant changes in ferritin in our study might indicate low or no inflammation in the study participants (since Peeling et al. [16] reported an increase in ferritin during exercise-induced inflammation), low or no oxidative stress [15] and minimal damage including damaged blood cells [8]. 

### 4.2. Changes in the mRNA of Genes Involved in Iron Metabolism 

The genes related to iron metabolism that were selected for analysis are easily induced by stressful conditions, and sensitive to intracellular iron concentrations, oxidative stress and hypoxia [30,31]. To the best of our knowledge, this is the first study in which changes in the mRNA of these genes were examined after a marathon run. The significant decrease in mRNA of *PCBP1* and *PCBP2* (expression partners) and *FTH* was observed post-race while at 3 h after the race an up-regulation occurred in *PCBP1* and *PCBP2* as well as in *FTH* and *FTL*. Furthermore, 24 h after the run the gene mRNA levels returned to baseline values. Unfortunately, discussion about these changes is hard since, as mentioned before, there are no data on this topic in the current literature. We assumed that the increase in the mRNA of genes involved in apoptosis and inflammatory response reported earlier [32] would cause long-term up-regulation in our tested genes i.e., that remained up-regulated 24 h after a marathon run. Unfortunately, this was not confirmed by our results. According to the literature, PCBP1 and PCBP2 proteins are iron chaperones that deliver iron to ferritin, the iron storage protein [17,33]. Thus, it is expected that an increase in the expression of these genes might play a protective role against iron toxicity. The mRNA levels of *TFRC* remained unchanged during the marathon run and in the recovery period (with a slight tendency to decrease compared to basal values) suggesting that the intracellular labile iron pool was kept under control. In turn, the *CAT* mRNA level decreased after the marathon run but also returned to baseline after 24 h. The results at 3 h after the marathon showed a significant increase in mRNA levels. It is established that during exercise, changes in many plasma or serum parameters influence intracellular homeostasis. Oxidative stress is another indicator of tested gene expression; thus, we evaluated the mRNA levels of C*AT* for additional information on changes in intracellular oxidative stress. One of the functions of catalase is an increase in antioxidative capacity (Sureda et al. [34]), thus its expression indirectly shows the level of oxidative stress in the cell. 

### 4.3. Relationship Between the mRNA of Genes Involved in Iron Metabolism and Running Speed

Generally, the same direction of changes in *PCBP1*, *PCBP2*, *FTH*, *FTL* and *TFRC* mRNA was observed in both groups, indicating a tendency to decrease immediately after the run and increase 3 h post-race. However, significant differences in *FTH* and *FTL* mRNA were observed between the slow and fast groups at 24 h after finishing the marathon. In faster participants, the mRNA levels of these genes were significantly lower compared to slower participants. According to Jastrzębski et al. [14], based on organ damage indicators, our findings could be caused by a better adaptation to a long-lasting effort in the faster group. The cited authors concluded that participants choose their running speed to individual possibilities determined by changes in tested parameters. In our opinion, the results obtained in our experiment, regarding changes in gene expression, indicating that this hypothesis could be true. Additionally, significant differences between groups (slow and fast) indicated that faster runners were significantly younger than slower runners. This finding indicated possibilities of influence of age to obtained results during marathon run. However, after dividing the participants of the run into two groups by the median split of age (younger and older), no significant differences between groups have been observed.

## 5. Conclusions

We concluded that marathon running induced changes in biochemical parameters and the expression of genes involved in iron metabolism, but these changes occurred at different time points. Interestingly, in faster runners, the return to basal values occurred faster than in slower runners. Generally, the amateurs could adjust the pace of the run to their capabilities. 

## Figures and Tables

**Table 1:** Characteristic of the participants. Data are presented as a range or means ± standard deviation (SD).

Participant’s Characteristic	
	Baseline (*n* = 24)
Age (years)	48.8 ± 6.5
Body mass (kg)	80.1 ± 8.5
Height (cm)	178.7 ± 5.3
BMI (kg/m^2^)	25.1 ± 2.3
PBF (%)	15.5 ± 5.0
Pace during the run (km/h)	10.9 ± 1.4
Training units per week	Between 1 and 7
Training regimen (km/week)	Between 20 and 115
Training experience (years)	Between 4 and 24
Number of finished marathons	Between 1 and 62

BMI—body mass index, PBF—percentage body fat.

**Table 2:** Changes in laboratory parameters after a marathon run (*n* = 24). *p* < 0.05 is statistically significant. Data are presented as the mean ± SD for the pre-race and the three following measurements. Values are corrected for plasma volume changes (%Delta PV). Statistical analyses were undertaken using Tukey’s multiple comparisons test for the parametric values and Dunn’s multiple comparisons test for nonparametric values, compared to the pre-race values. * *p* < 0.05.

	Pre-Race	Post-Race	3 h Post-Race	24 h Post-Race
%Delta PV	-	1.05%	11.58% *	12.43% *
	-	*p* > 0.9999	*p* < 0.0001	*p* < 0.0001
Hemoglobin (g/dL)	14.94 ± 0.85	14.78 ± 1.36	12.63 ± 1.06 *	12.41 ± 0.96 *
	-	*p* = 0.9545	*p* < 0.0001	*p* < 0.0001
RBC (×10^12^/L)	4.97 ± 0.33	4.91 ± 0.44	4.19 ± 0.35 *	4.13 ± 0.32 *
	-	*p* = 0.9445	*p* < 0.0001	*p* < 0.0001
Hematocrite (%)	43.33 ± 2.17	42.61 ± 3.96	35.91 ± 3.09 *	35.91 ± 2.58 *
	-	*p* > 0.9999	*p* < 0.0001	*p* < 0.0001
Reticulocytes (×10^9^/L)	59.44 ± 11.6	61.78 ± 13.77	49.38 ± 11.67 *	46.63 ± 12.52 *
	-	*p* = 0.9088	*p* = 0.0261	*p* = 0.0027
White blood cells (×10^9^/L)	5.27 ± 1.22	14.74 ± 3.45 *	12.02 ± 2.07 *	6.35 ± 1.54
	-	*p* < 0.0001	*p* < 0.0001	*p* = 0.9970
Neutrophils (×10^9^/L)	2.84 ± 0.9	12.2 ± 3.11 *	9.82 ± 1.97 *	3.58 ± 1.63
	-	*p* < 0.0001	*p* < 0.0001	*p* > 0.9999
Lymphocytes (×10^9^/L)	1.7 ± 0.31	1.48 ± 0.47 *	1.26 ± 0.28	1.94 ± 0.45
	-	*p* = 0.1485	*p* = 0.0007	*p* = 0.9166
Monocytes (×10^9^/L)	0.47 ± 0.14	0.95 ± 0.26 *	0.89 ± 0.21 *	0.56 ± 0.13
	-	*p* < 0.0001	*p* < 0.0001	*p* > 0.9999
Eosinophils (×10^9^/L)	0.22 ± 0.14	0.04 ± 0.04 *	0.01 ± 0.01 *	0.19 ± 0.12
	-	*p* < 0.0001	*p* < 0.0001	*p* > 0.9999
Basophils (×10^9^/L)	0.04 ± 0.02	0.06 ± 0.02 *	0.03 ± 0.01	0.04 ± 0.01
	-	*p* = 0.0032	*p* > 0.9999	*p* > 0.9999
CRP (mg/L)	1.4 ± 3.7	1.31 ± 3.26	1.87 ± 3.12	9.79 ± 7.28 *
	-	*p* > 0.9999	*p* = 0.0807	*p* < 0.0001
Uric acid (mg/dL)	5.26 ± 1.08	5.9 ± 1.07 *	5.71 ± 1.03 *	4.86 ± 0.96 *
	-	*p* < 0.0001	*p* = 0.0007	*p* = 0.0113
Creatine kinase (U/L)	171.56 ± 68.52	569.55 ± 490.71 *	871.04 ± 900.02 *	1410.66 ± 1444.06 *
	-	*p* < 0.0001	*p* < 0.0001	*p* < 0.0001

**Table 3:** Characteristics of slow and fast groups. Data are presented as means ± standard deviation (SD). * *p* < 0.05 for comparison between two groups.

Slow and Fast Groups Characteristics		
	Slow (*n* = 12)	Fast (*n* = 12)
Pace during the run (km/h)	10.04 ± 0.52	12.18 ± 0.71 *
Age (years)	53.58 ± 5.45	44.25 ± 3.49 *
BMI (kg/m^2^)	26.28 ± 1.88	23.83 ± 1.95 *
Training units per week	3.00 ± 1.04	4.58 ± 1.26
Training regimen (km/week)	43.58 ± 16.53	71.27 ± 25.22
Training experience (years)	10.83 ± 6.90	8.08 ± 5.02
Number of finished marathons	16.83 ± 20.85	13.58 ± 9.18
Baseline iron level (µg/dL)	112.75 ± 40.54	92.92 ± 42.44
Baseline ferritin level (ng/mL)	93.41 ± 36.58	88.18 ± 98.29
